# Evaluating the Efficacy of Active Ingredients Used in Roach Baits against Small Hive Beetle (*Aethina tumida*) and Their Safety to Honey Bees (*Apis mellifera*)

**DOI:** 10.3390/insects15070472

**Published:** 2024-06-25

**Authors:** Julia St. Amant, Amber Bisiau, Cameron Jack

**Affiliations:** Entomology and Nematology Department, University of Florida, Gainesville, FL 32611, USA; ambermoody@ufl.edu (A.B.); cjack@ufl.edu (C.J.)

**Keywords:** chemical control, abamectin, clothianidin, fipronil, hydramethylnon, indoxacarb

## Abstract

**Simple Summary:**

Small hive beetles (*Aethina tumida*) are a honey bee pest with few reliable in-hive chemical treatments available for beekeepers to use in the United States. Gel roach baits have been used off-label by commercial beekeepers as an alternative way to reduce small hive beetle populations. The objective of this study was to determine the toxicity of the active ingredients in gel roach baits to small hive beetles and honey bees (*Apis mellifera*) through topical exposure. In addition, we exposed small hive beetles to the active ingredients orally through pollen. All the active ingredients tested were more toxic to honey bees than to small hive beetles, except for fipronil, which was only slightly less toxic to honey bees than small hive beetles. The results of this study imply that gel roach baits should not be used in honey bee hives as small hive beetle treatments due to their toxicity to honey bees.

**Abstract:**

Beekeepers need new treatment options for controlling small hive beetles (*Aethina tumida*), a devastating honey bee (*Apis mellifera*) pest. For many years, commercial beekeepers in the U.S. have used gel roach baits off-label as a method for treating SHBs. Herein, we evaluated the acute toxicity of active ingredients commonly found in gel roach baits, including abamectin, clothianidin, hydramethylnon, fipronil, and indoxacarb through topical and oral routes of exposure against SHBs and honey bees. Additionally, coumaphos, the active ingredient of the only registered in-hive control treatment for SHBs, was evaluated to provide a comparison to the gel roach bait active ingredients. Fipronil was the most toxic compound to SHBs topically (LD_50_ = 0.23 ng/SHB) and through pollen (LC_50_ = 0.06 µg/g pollen). Fipronil (LD_50_ = 0.31 ng/honey bee) had a selectivity ratio of 1.3, suggesting that it is more toxic to SHBs than it is to honey bees, but only to a small degree. Abamectin, clothianidin, hydramethylnon, and indoxacarb had a higher toxicity to honey bees than to SHBs through topical exposure. Our results suggest that gel roach baits and their active ingredients are toxic to honey bees and pose a serious risk to colony safety if used as in-hive treatments.

## 1. Introduction

The small hive beetle (SHB), *Aethina tumida*, is one of the most destructive pests of honey bees (*Apis mellifera*). This pest is native to sub-Saharan Africa, but can now be found around the world including areas of North America, South America, Europe, and Australia [1]. Commercial beekeepers have been heavily impacted by SHBs since their introduction in the United States [2]. Small hive beetles damage resources and wax within a hive and in extreme infestations can cause honey bee colonies to abscond [3]. They can be especially problematic inside a honey house, destroying stored honey frames that were to be extracted [2]. Due to SHB damage to honey bee colonies and their production, commercial beekeepers certainly experience the negative financial impacts this pest has on their operations.

Chemicals are one of the methods used to control infestations of SHBs inside honey bee colonies. However, there is currently only one registered in-hive chemical control registered for use in the United States, coumaphos, applied under the trade name Checkmite+, which is an organophosphate insecticide. This class of chemical compounds act on the acetylcholinesterase, inhibiting the enzyme leading to the accumulation of acetylcholine, and inevitably the disruption of neurotransmission [4,5]. Unfortunately, SHBs have developed resistance to coumaphos in the United States, most likely due to enhanced detoxification by esterases and mixed-function oxidases [6]. This resistance places a limit on the type of pesticides that are successful in the reduction in beetles in the hive and the preservation of honey bees.

Many commercial beekeepers have found success in decreasing SHB populations in hives by creating their own treatments using corrugated cardboard applied with roach baits containing fipronil as an active ingredient [7]. Fipronil is a phenylpyrazole insecticide that works by inhibiting receptors on neurons in the central nervous system of insects and is incredibly toxic to honey bees relative to other chemicals [8,9]. Fipronil-containing gel baits and sprays have been used for decades to kill ants, roaches, and termites in homes [10,11]. Since finding that fipronil in roach baits was effective in killing the SHBs inside honey bee colonies, more beekeepers have been prompted to use fipronil-laced baits that are added to homemade traps [12]. Beekeepers have since started using other types of roach baits to control the small hive beetles in their bee populations. Currently, there are numerous cockroach baits on the market (Table 1) [11,13,14,15].

Herein, we conducted assays using the following active ingredients of various roach baits, namely: abamectin, clothianidin, fipronil, hydramethylnon, and indoxacarb. Abamectin is a type of avermectin-based insecticide that impacts insect peripheral nervous systems and their GABA receptors to block electrical activity [16]. This active ingredient is found in the gel bait product Avert^®^ DF Dry Flowable Cockroach Bait. Clothianidin is a neonicotinoid insecticide that targets the nicotinic acetylcholine receptor in the central nervous system to shut down electrical signals [17]. This active ingredient is found in the gel bait product Maxforce^®^ IMPACT Roach Gel Bait. Fipronil, described above, is found in the gel bait product COMBAT MAX ™ Roach Killing Bait. Hydramethylnon, the active ingredient in Maxforce^®^ Roach Killer Bait Gel, is a slow-acting insecticide classified as a mitochondrial electron transport inhibitor that impacts mitochondrial respiration [18]. Indoxacarb is an oxadiazine that impedes neuronal sodium channels, thus disrupting their nervous system [16]. This active ingredient is found in the gel bait product Advion^®^ Cockroach Gel Bait. The main objective of this study was to determine whether any of these active ingredients are capable of effective control of SHBs. Additionally, we want to better understand the impact that exposure of these chemicals could possibly have on honey bees when they become exposed inside a hive.

## 2. Materials and Methods

### 2.1. Small Hive Beetle Collection

Small hive beetles were collected from an in vitro-reared colony that were reared following the methods of Stuhl (2023) at the University of Florida Honey Bee Research and Extension Laboratory (UF HBREL). Within one week of emerging, SHBs of both sexes were transferred, by hand, into plastic cups (Uline 266 mL Squat Crystal Clear Plastic Cups and Flat Lids) with small ventilation holes as described in Kleckner et al. (2022) [12].

### 2.2. Honey Bee Collection

Frames of emerging adult worker honey bees were collected January–June 2023 at the UF HBREL (Entomology and Nematology Department, Gainesville, FL, USA, 29°38′4″ N, 82°21′57″ W) from optimally managed hives, meaning that an effort was made to keep *Varroa destructor* levels low and food resources high. No SHB or gel roach bait treatments had ever been used in these hives prior to this study to limit the possibility of the newly emerged bees having been exposed to any trace amounts of active ingredients of interest. The honey bees were brushed off and the frames were placed inside an incubation room at 32 °C to allow for adult honey bees to emerge. Less than 24 h later, the newly emerged honey bees from the frames were brushed and mixed together into a large bin. The honey bees were gently picked up by hand and placed into plastic cups (118.294 mL clear round wide-mouth plastic jar with white lid; ULINE S-9934) with small ventilation holes. Two 3 mL syringes with the tips cut off were hung through two holes in the top of the cups and filled with sucrose solution (1:1 *w*/*v*). The cups were placed in an incubator (Binder Incubator, (Camarillo, CA, USA, #BD400UL-120V) at 34 °C with no humidity control until the start of the assays that same day.

### 2.3. Topical Small Hive Beetle Assays

The three types of assays described in this paper were performed with the following compounds: abamectin (Sigma-Aldrich, St. Louis, MO, USA; 90% purity), clothianidin (Sigma-Aldrich; analytical standard), coumaphos (Sigma-Aldrich; 98% purity), fipronil (Chem Services, Inc., West Chester, PA, USA; 99.5% purity), hydramethylnon (Sigma-Aldrich; analytical standard), and indoxacarb (Sigma-Aldrich; 95% purity). Each assay contained a solvent control (acetone) and a positive control (dimethoate (MedChemExpress, Monmouth Junction, NJ, USA; 99% purity)) for comparison. Each treatment concentration was diluted with acetone from a high concentration stock solution (1000 µg/mL) of the solid compound mixed into acetone. Range finding to decide treatment groups was initially completed for each compound by using dilutions of ten from the stock solution.

For the SHB assays, the SHBs were anesthetized by spraying each cup with CO_2_ from a handheld CO_2_ tire inflator (Genuine Innovations Ultraflate Plus Inflator with 12 g Crosman Powerlet CO_2_ Cartridges) for approximately three seconds. The SHBs were topically dosed on their dorsal side with 1 µL of the solution using a micropipette (Eppendorf Research Plus, Single Channel Pipette, 0.5–10 µL). The SHBs were fed sucrose solution (1:1 *w*/*v*) that was placed into 1.5 mL microcentrifuge tubes with a wick made of braided cotton rolls (Richmond 10.16 cm, Medium Braided Cotton Roll) that allowed for SHB access without drowning. The cups were placed inside a desiccator (Fisherbrand Acrylic Desiccator Cabinets 45.7 cm 08-642-23C), containing a basin filled with tap water, located in incubators (Thermo Scientific Herather, IMH100 51028067 Bench Top Incubator, Lagenselbold, Germany) at 34 °C without humidity control. Pipette tips and gloves were changed between each chemical and each concentration change of each chemical. Mortality was checked every 24 h for 72 h [19].

### 2.4. Topical Bee Assays

Similar to Section 2.3, the honey bees were anesthetized with CO_2_ and topically dosed on the dorsal side of their thorax with 1 µL of the solution. Then, they were returned to their plastic cups and placed into an incubator (Binder Incubator, Hogentogler, Camarillo, CA, USA, #BD400UL-120V) at 34 °C. Pipette tips and gloves were changed between each chemical and each concentration change of each chemical. Mortality was checked every 24 h for 72 h, as per OECD protocol for acute topical assay for honey bees [19].

### 2.5. Pollen Small Hive Beetle Assays

Pollen patties were divided and rolled into 1 g pollen balls, wrapped loosely in plastic wrap, and placed into the freezer for future use. When used, the pollen was formed into small concave shapes before pipetting 50 µL of solution into each pollen ball as described in Kleckner et al. (2022) [12]. The pollen was then carefully mixed by hand to ensure a homogenous combination of the added solution throughout. The pollen was placed into the plastic cups with the small hive beetles, then the cups were placed into a desiccator (Fisherbrand Acrylic Desiccator Cabinets 45.7 cm 08-642-23C, Waltham, MA, USA), containing a basin filled with tap water, located in an incubator (Thermo Scientific Heratherm, IMH100 51028067 Bench Top Incubator, Lagenselbold, Germany) at 34 °C. Pipette tips and gloves were changed between each chemical and each concentration change of each chemical. The pollen balls remained in the cups for the duration of the experiment. Mortality was checked every 24 h for 72 h. 

### 2.6. Pollen Honey Bee Assays

As described in Section 2.5, pollen balls were mixed with 50 µL of solution by hand. Each cup containing ten honey bees received a pollen ball and the cups were placed into an incubator (Binder Incubator, Hogentogler, Camarillo, CA, USA, #BD400UL-120V) at 34 °C. Pipette tips and gloves were changed between each chemical and each concentration change of each chemical. The pollen ball remained in the cups for the duration of the experiment. Mortality was checked every 24 h for 72 h.

### 2.7. Statistical Analysis

Statistical analysis was performed using R version 4.2.1 (2022-06-23) “Funny-Looking Kid” using an R script for probit analysis of insects [20]. A heterogeneity factor was added as needed. The analysis was separated by compound and assay type to create individual LD_50_/LC_50_ values using a 95% confidence interval. Each analysis included the mortality after 72 h, the number of SHBs/bees tested, and the treatment concentrations. Assays that had a greater than 25% solvent control mortality or less than 90% positive control mortality were removed from the analysis and Abbott’s correction was calculated by R when mortality in the solvent control was greater than 5% [20]. The selectivity ratio for topical application was calculated by dividing the honey bee LD_50_ by the SHB LD_50_ of each compound. The activity ratio for each compound was calculated by dividing the LD_50_/LC_50_ of coumaphos, the industry standard, with the LD_50_/LC_50_ of the compound within each assay type.

## 3. Results

### 3.1. Topical Small Hive Beetle Assays

Among the compounds treated topically on SHBs, fipronil was the most toxic and had the lowest LD_50_ value (LD_50_ = 0.23 ng/SHB; Table 2). Hydramethylnon was the least toxic compound and had the highest LD_50_ value (LD50 = 1136.36 ng/SHB), being 2.6× less toxic than coumaphos. Clothianidin (LD_50_ = 35.35 ng/SHB) was the second most toxic compound tested but was only 12× more toxic than coumaphos. Abamectin (LD_50_ = 97.52 ng/SHB) and indoxacarb (LD_50_ = 215.22 ng/SHB) were 4× and 2× more toxic than coumaphos, respectively. More than 90% of the SHBs treated topically with dimethoate (1000 ng/SHB) died within 72 h of exposure (Table S1).

### 3.2. Topical Honey Bee Assays

Abamectin had the lowest LD_50_ value (LD_50_ = 0.12 ng/honey bee) on honey bees through topical exposure and was 50,000× more toxic than coumaphos (LD_50_ = >6000 ng/honey bee) (Table 3). Coumaphos was unable to produce >50% mortality from doses up to 6000 ng/bee. Hydramethylnon (LD_50_ = 112.32 ng/honey bee) was the least toxic novel compound to honey bees and was still 53× more toxic than coumaphos. Fipronil (LD_50_ = 0.31 ng/honey bee), clothianidin (LD_50_ = 5.50 ng/honey bee), and indoxacarb (LD_50_ = 12.05 ng/honey bee) were 19,355×, 1091×, and 498× more toxic to honey bees than coumaphos, respectively. More than 90% of the honey bees treated with dimethoate (1000 ng/honey bee) died within 72 h of exposure (Table S1).

We directly compared the LD_50_ values between the topical assays of the SHBs and the honey bees, as these assays were the same for both animals. Based on the selectivity ratios (Table 3), both fipronil and coumaphos were more toxic to SHB than to honey bees, being 1.3× and >14× more toxic, respectively. However, abamectin (813×), clothianidin (6×), hydramethylnon (10×), and indoxacarb (18×) were all more toxic to honey bees than they were to SHBs.

### 3.3. Pollen Small Hive Beetle Assays

Two of the novel compounds, abamectin and hydramethylnon (LC_50_ = >200 µg/g pollen), did not produce SHB mortality >50% from doses up to 200 µg/g pollen (Table 4). Fipronil had the lowest LC_50_ value (LC_50_ = 0.06 µg/g pollen), which was 2917× more toxic than coumaphos (LC_50_ = 175.01 µg/g pollen). Clothianidin (LC_50_ = 5.87 µg/g pollen) and indoxacarb (LC_50_ = 26.26 µg/g pollen) were 30× and 7× more toxic to SHBs than coumaphos, respectively (Table S1).

### 3.4. Pollen Honey Bee Assays

The honey bees would not consistently interact or consume the treated pollen within the 72 h period. Multiple adjustments were made to the pollen balls to encourage the honey bees to feed on them, such as the introduction of powdered sugar inside and outside the pollen ball. Despite these attempts, the pollen remained mostly untouched, and the positive controls (dimethoate) did not produce high enough mortality (<90%) to trust our findings with this assay. Therefore, the toxicity of the test compounds consumed by honey bees was considered invalid.

## 4. Discussion

The purpose of this study was to evaluate the efficacy of active ingredients used in roach bait gels, which are often used off-label inside honey bee colonies to control SHB infestations and test their acute impact on honey bee health. Our data revealed that coumaphos, the only in-hive chemical currently registered for SHB control in the United States, was ineffective at killing SHBs. Thus, we have confirmed findings from Kanga et al. (2021) [6] that SHBs are certainly resistant to coumaphos in the United States. We found that abamectin, clothianidin, hydramethylnon, and indoxacarb were all more toxic to honey bees than they are to SHBs, making them poor options for in-hive SHB control. While fipronil exhibited slightly higher toxicity towards SHBs compared to honey bees, the marginal difference in toxicity levels to both animals make it a highly dangerous compound when used within honey bee hives.

It is unfortunately common for pesticides to be used off-label in honey bee hives to treat unwanted pests. However, introduction of these pesticides into a hive could have detrimental impacts to the colony if the bees are able to come into contact with them. Though commercial beekeepers may attempt to separate honey bees from the roach bait using corrugated cardboard with holes only big enough for SHBs to reach the bait, if these cardboard strips were to malfunction or the SHB was able to move the roach bait outside of the cardboard strip as suggested by Kleckner et al. [12], then the whole colony would be at risk. Ideally, SHB treatments would have a high selectivity ratio, meaning they can be introduced into colonies at levels low enough to be safe for honey bees, but are still high enough to decrease SHB populations.

We also explored the toxicity of roach bait active ingredients to SHBs through pollen. This route of exposure could be used in future SHB treatment delivery systems [12], and is a hybrid between oral and topical exposure, as the SHBs would crawl in and around the pollen, sometimes laying eggs. A previous study showed that SHBs exposed to fipronil in the hive using compact disc cases to exclude honey bees did not die immediately after exposure. It was likely that the compound was transported to other parts of the hive after exposure, which increased the risk of exposure to honey bees [12]. This implies that roach bait gels can potentially be spread around the hive even when applied to an isolated area. It is possible that the SHBs in our assay were only consuming the insecticide-treated pollen because they were not provided any other food sources for the 72 h period. When we compared the SHB LC_50_ values of these compounds to the SHB LC_50_ of coumaphos, the only registered in-hive treatment for SHBs, we found that fipronil, clothianidin, and indoxacarb were 2917×, 30×, and 7× more toxic than coumaphos, respectively. Hydramethylnon and abamectin were less effective than coumaphos. This oral toxicity information is useful when considering active ingredients as candidates for possible treatments to be developed in the future.

Pollen tests were conducted on honey bees using the same methods, but were unsuccessful, which unfortunately prevents selectivity ratios from being created to compare SHB and honey bee LC_50_s for pollen exposure. Peak pollen consumption for honey bees is between 4 and 9 days post-emergence as an adult [21]. The honey bees used this study were fed pollen the same day they emerged from their cells and may have been too young to actively consume the pollen. Ten-day chronic honey bee oral assays may be a more suitable test option in the future, as they would allow the honey bees more time to ingest the pollen. Honey bee oral assays are often conducted using sucrose solutions to induce acute oral toxicity [22]. However, honey bees within hives that are treated with these active ingredients would likely be exposed through pollen patties instead of sucrose. Exposing SHBs to active ingredients through pollen has been shown to be a successful method of treatment [12] and would probably be the most likely delivery method in future treatments. Therefore, the LC_50_ value for acute oral toxicity in honey bees through sucrose may not be as relevant to future development of an SHB treatment as the LC_50_ of chronic oral toxicity through pollen.

## 5. Conclusions

Overall, roach bait gels are a risky and illegal treatment option against SHBs due to their toxicity to honey bees. Our study evaluated acute exposure, which focuses on adult honey bee mortality due to short-term exposure of highly concentrated pesticides in a laboratory setting. The roach bait active ingredients evaluated in this study were not an effective or safe option to be used against SHBs. The chronic impacts on honey bees, acute impact on brood, and overall impact on hive health were not tested here but would provide a more holistic understanding of how gel roach baits impact honey bee colonies. Furthermore, future research should be conducted to find active ingredients that are more selective to SHBs.

## Figures and Tables

**Table 1:** Detail of commercial gel cockroach baits.

Active Ingredient	% of Active Ingredient	Trade Name
Abamectin	0.050	Avert^®^ DF Dry Flowable Cockroach Bait
Clothianidin	1.0	Maxforce^®^ IMPACT Roach Gel Bait
Fipronil	0.03	COMBAT MAX ™ Roach Killing Bait
Hydramethylnon	0.01	Maxforce^®^ Roach Killer Bait Gel
Indoxacarb	0.6	Advion^®^ Cockroach Gel Bait

**Table 2:** Lethal dose (LD_50_) values at 72 h post-treatment and confidence intervals for small hive beetle acute topical toxicity generated in R. The activity ratio (AR) = LD_50_ of SHBs with coumaphos/LD_50_ of SHBs with the test compound. The activity ratio (AR) comparing each active ingredient with coumaphos is included.

Compound	SHBs (n)	SHBs LD_50_ (95% CI)	AR
Coumaphos	270	431.37 ng/SHB (394.0–437.49)	--
Fipronil	349	0.23 ng/SHB (0.11–0.51)	1876
Clothianidin	300	35.35 ng/SHB (6.94–114.56)	12
Abamectin	288	97.52 ng/SHB (62.52–120.03)	4
Indoxacarb	240	215.22 ng/SHB (47.87–312.02)	2
Hydramethylnon	181	1136.36 ng/SHB (1061.85–1297.83)	0.38

**Table 3:** Lethal dose (LD_50_) values at 72 h post-treatment and confidence intervals for honey bees’ acute topical toxicity, generated in R. The activity ratio (AR) = LD_50_ of honey bees with coumaphos/LD_50_ of honey bees with the test compound. The selectivity ratio (SR) is equal to the topical LD_50_ of the honey bee divided by the LD_50_ of the small hive beetle (reported in Table 2) for each compound.

Compound	Honey Bees (n)	Honey Bees LD_50_ (95% CI)	AR	SR
Coumaphos	210	>6000 ng/honey bee	--	>14
Abamectin	361	0.12 ng/honey bee (0.02–0.24)	<50,000	813
Fipronil	149	0.31 ng/honey bee (0.18–0.60)	<19,355	1.3
Clothianidin	271	5.50 ng/honey bee (4.73–6.17)	<1091	6
Indoxacarb	210	12.05 ng/honey bee (11.05–13.05)	<498	18
Hydramethylnon	240	112.32 ng/honey bee (29.85–185.78)	<53	10

**Table 4:** Lethal concentration (LC_50_) values at 72 h post-treatment and confidence intervals for the toxicity of compounds to small hive beetles through pollen, generated in R. The activity ratio (AR) = LC_50_ of SHBs with coumaphos/LC_50_ of SHBs with the test compound.

Compound	SHBs (n)	SHBs LC_50_ (95% CI)	AR
Coumaphos	362	175.01 µg/g pollen (108.75–457.12)	--
Fipronil	300	0.06 µg/g pollen (0.04–0.10)	2917
Clothianidin	180	5.87 µg/g pollen (0.47–13.05)	30
Indoxacarb	391	26.26 µg/g pollen (4.91–54.79)	7
Abamectin	210	>200 µg/g pollen	>0.875
Hydramethylnon	240	>200 µg/g pollen	>0.875

## Data Availability

The raw data supporting the conclusions of this article will be made available by the authors on request.

## Supplementary Materials

The following supporting information can be downloaded at: https://www.mdpi.com/article/10.3390/insects15070472/s1, Table S1: The treatment concentration, survival percentage, and mortality percentage for each active ingredient used in the small hive beetle and honey bee assays.
