# Supplementary material for: Evaluating the Efficacy of Active Ingredients Used in Roach Baits against Small Hive Beetle (Aethina tumida) and Their Safety to Honey Bees (Apis mellifera)
Source: Insects. 2024 Jun 25;15(7):472. doi: 10.3390/insects15070472 (PMC11277340; doi:10.3390/insects15070472)
Supplement: Supplementary file 1 [file insects-15-00472-s001.zip › insects-2989927-supplementary.pdf]

Supplementary Table S1. The treatment concentration, survival percentage, and mortality percentage for each active ingredient used in the small hive beetle and honey bee assays.

SHB Topical Assays

Compound	Concentration (µg/ml)	Survival (%)	Mortality (%)
Controls	Acetone	96	4
	Dimethoate (1000 µg/ml)	0	100
Coumaphos	100	100	0
	250	90	10
	300	76.7	23.3
	400	66.7	33.3
	500	43.3	56.7
	750	3.3	96.7
	1000	0	100
	2000	0	100
	3000	0	100
Fipronil	0.01	100	0
	0.025	90	10
	0.05	83.3	16.7
	0.1	70	30
	0.25	74.3	25.7
	0.5	21.4	78.6
	0.75	3.6	96.4
	1	0	100
Abamectin	50	83.3	16.7
	100	73.3	26.7
	110	66.7	33.3
	120	14.3	85.7
	125	10	90
	150	13.3	86.7
	200	0	100
	250	0	100
	500	0	100
	750	0	100
Clothianidin	0.1	96.67	3.33
	1	100	0
	10	86.7	13.3
	25	45	55
	50	26.7	73.3
	75	43.3	56.7
	100	6.7	93.3
	1000	0	100
Hydramethylnon	1000	80	20

	1050	96.67	3.33
	1100	80	20
	1150	40	60
	1200	19.4	80.6
	1400	0	100
Indoxacarb	100	83.3	16.7
	200	80	20
	250	63.3	36.7
	300	10	90
	400	3.3	96.7
	500	10	90
	1000	0	100

Pollen SHB Assays

Compound	Concentration (μg/ml)	Survival (%)	Mortality (%)
Controls	Acetone	97.7	2.3
	Dimethoate (1000 μg/ml)	6.3	93.7
Coumaphos	500	100	0
	1000	78.3	21.7
	1250	93.3	6.7
	1500	100	0
	1750	76.7	23.3
	2000	93.3	6.7
	3000	68	32
	4000	38.7	61.3
	4500	75	25
	5000	0	100
	10000	0	100
Fipronil	0.1	100	0
	0.2	93.3	6.7
	0.3	90	10
	0.5	90	10
	1	40	60
	5	23.3	76.7
	10	1.7	98.3
	100	0	100
	1000	0	100
Abamectin	0.1	100	0
	1	100	0
	10	96.7	3.3
	100	96.7	3.3
	1000	100	0
	2000	90	10

	4000	73.3	26.7
Clothianidin	0.1	93.3	6.7
	1	93.3	6.7
	10	93.3	6.7
	50	60	40
	100	50	50
	250	43.3	56.7
	500	16.7	83.3
	750	20	80
	1000	0	100
Hydramethylnon	0.1	100	0
	1	100	0
	10	99.97	0.03
	100	100	0
	1000	100	0
	1500	100	0
	2000	100	0
	4000	100	0
Indoxacarb	1	100	0
	10	100	0
	100	100	0
	250	60	40
	500	66.7	33.3
	750	43.3	56.7
	1000	26.7	73.3
	1250	50	50
	1500	6.7	93.3
	2000	10	90
	4000	0	100
	6000	0	100

Honey Bee Topical Assays

Compound	Concentration (µg/ml)	Survival (%)	Mortality (%)
Controls	Acetone	92.5	7.5
	Dimethoate (1000 µg/ml)	0	100
Coumaphos	100	90	10
	500	96.7	3.3
	1000	76.7	23.3
	2000	76.7	23.3
	6000	80	20
	8000	46.7	53.3
	10000	40	60
Fipronil	0.1	96.7	3.3

	0.2	93.3	6.7
	0.25	66.7	33.3
	0.5	0	100
	0.75	10	90
Abamectin	0.01	80	20
	0.25	50	50
	0.3	13.3	86.7
	0.4	9.7	90.3
	0.5	0	100
	0.75	0	100
Clothianidin	2.5	86.7	13.3
	5	60	40
	6	43.3	56.7
	7.5	20	80
	8	42	58
	9	16.7	83.3
	10	23.3	76.7
	15	3.3	96.7
	20	0	100
Hydramethylnon	10	100	0
	25	90	10
	100	63.3	36.7
	150	42.6	57.4
	175	51.7	48.3
	200	3.3	96.7
	250	10	90
Indoxacarb	4	100	0
	5	100	0
	10	57.4	42.6
	12.5	56.7	43.3
	20	3.4	96.6
	30	0	100
	40	0	100
